# Efficacy of Transcranial Direct Current Stimulation for Chronic Non-Specific Low Back Pain: A Systematic Review and Meta-Analysis

**DOI:** 10.3390/healthcare14121764

**Published:** 2026-06-18

**Authors:** Yuchen Zhou, Yifei Shou, Siyao Qiu, Sitong Wang, Chuang Gao, Junqi Jia, Zhiqiang Liang, Min Xia

**Affiliations:** 1 Faculty of Sports Science, Ningbo University, Ningbo 315211, China; 236004278@nbu.edu.cn (Y.Z.); 236000323@nbu.edu.cn (Y.S.); 226004861@nbu.edu.cn (S.Q.); wstcy1110@foxmail.com (S.W.); nbugaochuang@outlook.com (C.G.); nbujiajunqi@outlook.com (J.J.); 2 School of Physical Education, Shanghai University of Sport, Shanghai 200438, China

**Keywords:** transcranial direct current stimulation, chronic non-specific low back pain, systematic review, meta-analysis

## Abstract

**Background**: Chronic non-specific low back pain (CNSLBP) is highly disabling, and the efficacy of transcranial direct current stimulation (tDCS) remains uncertain. This systematic review and meta-analysis evaluated the effects of standalone and combined tDCS on pain and functional disability in adults with CNSLBP, with secondary comparisons between M1 and DLPFC stimulation. **Methods**: PubMed, Web of Science, Science Direct, China National Knowledge Infrastructure, Chinese Quarterly Virtual Information Platform, and Wanfang Data were searched up to February 2026. Randomized controlled trials of standalone or combined tDCS in adults with CNSLBP were included. Meta-analyses assessed pain intensity and functional disability, with subgroup analyses comparing stimulation targets and meta-regression exploring protocol parameters. Evidence certainty was evaluated using GRADE, and the protocol was registered with PROSPERO (CRD420261341095). **Results**: Meta-analysis of 17 trials showed that standalone tDCS did not significantly reduce pain intensity, whereas tDCS combined with peripheral interventions yielded a significant analgesic effect, and anodal stimulation over M1 significant reduced pain intensity. However, neither standalone nor combined tDCS significantly improved functional disability, subgroup differences between M1 and DLPFC were non-significant, and meta-regression identified no significant moderators. Descriptive findings showed potential benefits of tDCS for pain catastrophizing and postural control, with mild adverse events. **Conclusions**: Moderate-certainty evidence suggests that standalone tDCS is unlikely to provide clinically meaningful analgesia for CNSLBP. Low-certainty evidence indicates that anodal M1 tDCS combined with structured peripheral interventions, particularly exercise, may produce analgesic benefits, but this finding requires cautious interpretation.

## 1. Introduction

Chronic non-specific low back pain (CNSLBP) is a leading cause of disability and work incapacity worldwide, imposing a substantial socioeconomic burden on modern healthcare systems [1,2]. Although conventional conservative treatments—including pharmacological interventions, various forms of exercise rehabilitation, and cognitive behavioral therapy—are widely employed in clinical practice, long-term outcomes for most patients remain limited, and recurrence rates are notably high [3]. Recent evidence suggests that combined exercise modalities, such as aerobic exercise paired with core stabilization training, may yield additional functional benefits in specific subpopulations [4], yet the overall efficacy of conservative approaches remains suboptimal. Consequently, the exploration of more effective therapeutic strategies has remained a research priority in this field.

In recent years, advancements in neuroimaging and electrophysiology have reshaped our understanding of the pathophysiological mechanisms underlying CNSLBP. Accumulating evidence indicates that the persistence and exacerbation of CNSLBP are not only attributable to sustained nociceptive input from the periphery but is also closely associated with maladaptive plasticity within the central nervous system [5,6]. Meanwhile, central sensitization, characterized by altered excitability of the primary motor cortex (M1) and dysfunction of the descending pain inhibitory network, is now widely recognized as a core mechanism underlying the development and maintenance of chronic low back pain [7,8]. This paradigm shift in mechanistic understanding has directed research attention toward novel interventions capable of directly modulating central nervous system function.

Transcranial direct current stimulation (tDCS), a promising noninvasive brain stimulation protocol, provides a feasible approach to meet the therapeutic needs outlined above [9]. By delivering a weak constant current through scalp electrodes, tDCS modulates the resting membrane potential of cortical neurons in a polarity-dependent manner, thereby altering local cortical excitability [9]. Theoretically, positioning the anode over key targets such as M1 or the dorsolateral prefrontal cortex (DLPFC) can activate “top-down” descending pain inhibitory pathways, and potentially reverse maladaptive neuroplasticity induced by chronic pain [10,11]. Leveraging these mechanistic advantages, tDCS has been progressively investigated in clinical trials for CNSLBP emerging as a research hotspot in this field [12].

Although tDCS has a clear mechanistic rationale at the theoretical level, the clinical evidence from existing trials remains strikingly inconsistent. While some randomized controlled trials have reported beneficial effects of tDCS on pain relief and physical function improvement [13,14], others have failed to observe significant clinical benefits superior to sham stimulation [15,16], resulting in considerable heterogeneity and debate within the existing evidence base. For instance, a recent meta-analysis by Yang et al. (2020) pooled studies without distinguishing between standalone and combined tDCS protocols or stratifying by stimulation target [17], which may have obscured differential effects arising from distinct application modalities and cortical targets. A critical analysis reveals two key reasons for this controversy. First, previous studies have often conflated two fundamentally distinct application modalities, namely tDCS as a standalone therapy versus tDCS as an adjunct to peripheral rehabilitation, without proper distinction [18]. In practice, tDCS can be administered either as a standalone intervention or in combination with peripheral rehabilitation approaches such as exercise therapy and pain neuroscience education [19,20], and the therapeutic effects of these two modalities may be inherently different. Second, most prior systematic reviews have focused narrowly on pain intensity as the sole outcome measure [17], neglecting the multidimensional nature of CNSLBP, which encompasses sensory, cognitive, psychological, and physiological domains [21]. Consequently, the modulatory effects of tDCS on pain-related psychological states (e.g., catastrophizing, kinesiophobia), cognitive executive functions, postural control, and neurophysiological markers remain insufficiently synthesized and evaluated through systematic quantitative evidence. For instance, although one systematic review has focused on the effects of tDCS on depressive symptoms [22], systematic evaluations of other dimensions remain scarce.

To address these knowledge gaps, this systematic review and meta-analysis was designed with a prespecified hierarchy of objectives. The primary objective was to compare the analgesic and functional effects of standalone tDCS versus tDCS combined with peripheral rehabilitation interventions. The secondary objective was to compare the effects of M1 versus DLPFC stimulation through prespecified subgroup analyses. An exploratory objective was to systematically synthesize the modulatory effects of tDCS on multidimensional outcomes, including psychological states, cognitive function, postural control, and neurophysiological markers. This hierarchical structure was designed to first establish whether the addition of interventions modifies tDCS efficacy, and then to examine whether the stimulation target further influences outcomes within each application modality.

## 2. Materials and Methods

### 2.1. Study Design and Registration

This study is a systematic review and meta-analysis. The protocol was developed, and the report is structured, in strict accordance with the PRISMA 2020 statement guidelines [23,24]. The protocol was prospectively registered with PROSPERO (registration number: CRD420261341095).

### 2.2. Inclusion and Exclusion Criteria

#### 2.2.1. Inclusion Criteria

Studies were selected according to the PICOS framework as outlined below:

Population (P): adult patients aged ≥18 years with a definitive clinical diagnosis of CNSLBP. This requires the presence of lumbar pain for a duration exceeding three months, with no identifiable specific pathological cause (e.g., infection, malignancy, fracture, inflammatory diseases such as ankylosing spondylitis, or definitive nerve root compression symptoms); Intervention (I): tDCS, including conventional tDCS or high-definition transcranial direct current stimulation (HD-tDCS), employed as the core therapeutic modality. tDCS administered either as a standalone therapy or as a combined intervention integrated with other treatments (e.g., exercise therapy, physical therapy, cognitive behavioral therapy) was permitted; Comparison (C): the control group received sham tDCS (i.e., electrode placement identical to active stimulation, with current ramped up and down only at the beginning and end of the session to maintain blinding, without sustained effective stimulation), no intervention (blank control), conventional conservative treatment, or an active control regimen matched for any cointerventions administered to the experimental group; Outcomes (O): the primary outcome is the change in self-reported pain intensity from baseline to the end of the intervention, assessed using validated instruments such as the Visual Analogue Scale (VAS) or Numerical Pain Rating Scale (NPRS). Secondary outcomes are: (1) low back pain related functional disability, assessed using tools like the Oswestry Disability Index or Roland–Morris Disability Questionnaire; (2) pain-related psychological states, including pain catastrophizing (e.g., Pain Catastrophizing Scale), kinesiophobia (e.g., Tampa Scale for Kinesiophobia), anxiety (e.g., Hospital Anxiety and Depression Scale-Anxiety subscale; State–Trait Anxiety Inventory, STAI; Generalized Anxiety Disorder-7), and depression (e.g., Patient Health Questionnaire-9; Self-Rating Depression Scale); (3) quantitative sensory testing, such as pressure pain thresholds; (4) cognitive function assessments; (5) changes in brain function indicators; (6) health-related quality of life, assessed using instruments like the Short Form-36 or EuroQol five-dimension questionnaire; (7) patient-reported global impression of improvement or satisfaction; (8) safety indicators: adverse event rates and patient tolerability. Study design (S): randomized controlled trials, including both parallel-group and cross-over designs, published in peer-reviewed journals. The language was restricted to Chinese or English. No mandatory restrictions were applied regarding the minimum sample size per group or the minimum follow-up duration.

#### 2.2.2. Exclusion Criteria

Studies meeting any of the following criteria were excluded:

(1) Studies whose population explicitly included patients with chronic low back pain attributable to specific etiologies (e.g., neuropathic pain, fibromyalgia syndrome, pain secondary to radiculopathy); (2) Interventions involving other forms of peripheral electrical stimulation, invasive brain stimulation, or other non-invasive non-electrical brain stimulation protocols (e.g., repetitive transcranial magnetic stimulation, repetitive transcranial magnetic stimulation; transcranial alternating current stimulation); (3) Publication types lacking complete original data, such as conference abstracts, study protocols, systematic reviews or narrative reviews, letters, case reports, dissertations, or book chapters; (4) Studies for which the full text was unavailable or where critical outcome data were missing and could not be obtained after contacting the corresponding authors.

### 2.3. Literature Search Strategy

A systematic literature search was conducted independently by two reviewers across the following six major electronic databases, encompassing both English and Chinese sources: PubMed, Web of Science, Science Direct, the Chinese Quarterly Virtual Information Platform, the China National Knowledge Infrastructure, and the Wanfang Data knowledge service platform. The search period was defined from the inception of each database up to 7 February 2026. The search strategy employed a combination of Medical Subject Headings (MeSH) and free-text keywords, adapted to the specific search syntax of each database. Core English search terms included: “transcranial direct current stimulation”, “tDCS”, “HD-tDCS”, “transcranial electrical stimulation”, “low back pain”, “chronic low back pain”, and “non-specific low back pain”. The detailed search strings for each database are provided in Table S1. Furthermore, to minimize the risk of missing relevant studies, the reference lists of all finally included articles and pertinent previously published systematic reviews were manually screened by the two reviewers. No restrictions were applied regarding the year of publication.

### 2.4. Study Selection and Data Extraction

All records retrieved from electronic searches were imported into EndNote X9 (Clarivate Analytics, Philadelphia, PA, USA) for duplicate removal. Study selection was conducted independently by two trained reviewers in a two-step process: titles and abstracts were first screened to exclude clearly ineligible studies, after which full texts of potentially relevant articles were assessed against the predefined PICOS criteria. Disagreements were resolved through discussion, or by consultation with a third senior reviewer if consensus was not reached.

Data extraction was performed independently by two reviewers using a pilot-tested, standardized form. The extracted information included:

(1) Study Characteristics: first author, year, country, study design, sample size, and baseline patient characteristics (age, sex distribution, pain duration).
(2) Intervention Protocol: tDCS type, cortical target, electrode parameters (size, polarity, montage), current intensity (mA), session duration (min), frequency and total sessions, and whether combined with other therapies.
(3) Control Group: type of control group (e.g., sham stimulation, no intervention, conventional treatment).
(4) Outcome and Safety Data: mean values, standard deviations, and corresponding sample sizes at each reported time point; types and incidence rates of adverse events; dropout rates and reasons for attrition. For crossover randomized controlled trials, data were extracted only from the first intervention to minimize carryover effects, unless an adequate washout period was confirmed and paired analysis results were reported. When original articles had missing data or reported outcomes in a format unsuitable for meta-analysis, corresponding authors were contacted to request the necessary data. If no response was received, the study was included only in the descriptive qualitative synthesis and excluded from quantitative meta-analysis.

### 2.5. Assessment of Risk of Bias

Two reviewers independently assessed the methodological quality of the included randomized controlled trials using the Cochrane risk-of-bias tool for randomized trials (RoB 2) [25]. The assessment covered the following five domains: bias arising from the randomization process; bias due to deviations from intended interventions; bias due to missing outcome data; bias in measurement of the outcome; and bias in selection of the reported result. Each domain was judged as presenting a “low risk of bias,” raising “some concerns,” or presenting a “high risk of bias.” An overall risk-of-bias judgment for each study was determined according to the RoB 2 algorithm. Disagreements during the assessment process were resolved through discussion, with arbitration by a third reviewer if necessary. The results of the risk of bias assessment are reported in Section 3.2.

### 2.6. Certainty of Evidence Assessment

The certainty of evidence for each outcome was assessed using the Grading of Recommendations Assessment, Development and Evaluation (GRADE) framework [26], as recommended by the PRISMA 2020 statement [23,24]. Two reviewers independently rated the evidence across five domains: risk of bias, inconsistency, indirectness, imprecision, and publication bias. Each domain was judged as presenting no serious concerns, serious concerns (downgrade by one level), or very serious concerns (downgrade by two levels). The certainty of evidence was classified as high, moderate, low, or very low. Disagreements were resolved through consensus. The following comparisons were assessed: (1) standalone tDCS versus sham for pain intensity; (2) combined tDCS (M1) versus control for pain intensity; (3) combined tDCS (DLPFC) versus control for pain intensity; (4) standalone tDCS versus sham for functional disability; (5) combined tDCS (M1) versus control for functional disability; and (6) combined tDCS (DLPFC) versus control for functional disability.

### 2.7. Data Synthesis and Meta-Analysis

All quantitative data synthesis and meta-analyses were performed using R software (version 4.3.1) with the meta package (version 6.5.0) [27]. Given that the outcome measures in this study were all continuous variables, the mean difference (MD) with its 95% confidence interval (CI) was selected as the effect measure when different studies used identical scales to measure the same clinical outcome. If different instruments were employed to assess the same clinical outcome, the standardized mean difference (SMD) with its 95% CI was used for data pooling. Statistical heterogeneity among the included studies was quantified using Cochran’s Q test and the I^2^ statistic. When I^2^ ≤ 50% and the *p*-value from the Q test was >0.10, a fixed-effect model was applied for pooling. Conversely, if I^2^ > 50% or *p* ≤ 0.10, a random-effects model was employed to combine effect sizes, after a thorough investigation to rule out significant clinical or methodological heterogeneity as the primary cause. To further explore potential sources of heterogeneity in the combined tDCS analysis, univariate meta-regressions were performed with analgesic effect size (SMD) as the dependent variable and current intensity, total number of treatment sessions, and single-session duration as independent variables. Meta-regressions were conducted only when at least ten studies were available for the analysis. For studies that could not be included in the quantitative meta-analysis due to incomplete original data reporting, highly specific outcome measures, or excessive heterogeneity, a narrative synthesis approach was adopted to provide an objective descriptive summary of their main findings.

## 3. Results

### 3.1. Study Selection

**Figure 1:**
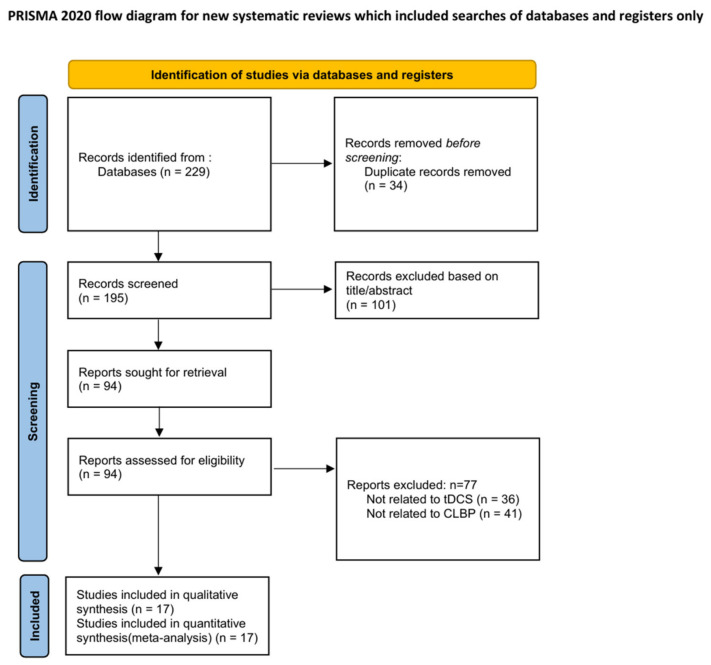
Literature screening flow chart.

The study selection process followed the PRISMA guidelines. An initial search of the databases yielded 389 records. After removing 112 duplicates, the titles and abstracts of the remaining 277 articles were screened, leading to the exclusion of 182 records. The full texts of the remaining 95 articles were assessed for eligibility, of which 76 were excluded for not meeting the inclusion criteria. Ultimately, 17 trials were included in this systematic review. The study selection process is illustrated in Figure 1. Of the 17 included RCTs, only one study [28] employed high-definition tDCS (HD-tDCS) targeting the medial prefrontal cortex. Given that only a standalone HD-tDCS study was available, no comparison between conventional tDCS and HD-tDCS could be performed.

### 3.2. Results of Risk of Bias Assessment

**Figure 2:**
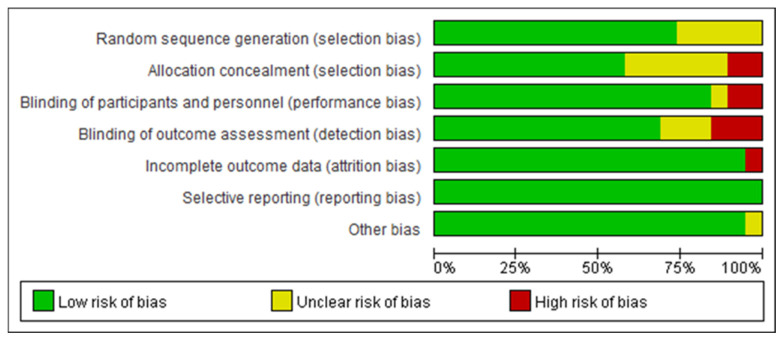
Risk of bias assessment results.

The risk of bias assessment for the 17 included randomized controlled trials is summarized in Figure 2. Overall, the majority of studies were judged as raising “some concerns” primarily in domains related to deviations from intended interventions and measurement of the outcome. No study was rated as having a high overall risk of bias.

### 3.3. Effect of tDCS on Pain Intensity

#### 3.3.1. Effect of Standalone tDCS on Pain Intensity

**Figure 3:**
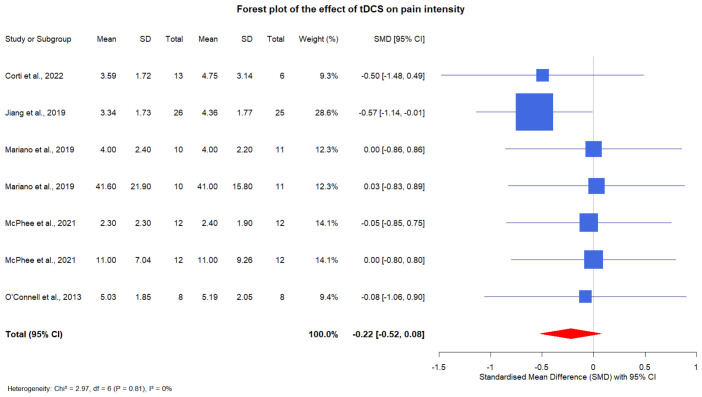
Forest plot of the effect of tDCS on pain intensity [15,16,28,29,30]. Blue squares denote the SMD of individual studies, with horizontal lines representing 95% confidence intervals; the size of each square corresponds to study weight. The red diamond indicates the pooled overall SMD and its 95% CI.

A total of five randomized controlled trials [15,16,28,29,30] comprising 176 participants (tDCS: 91; sham: 85) were included in the analysis (Figure 3). Using a fixed-effects model, the pooled results showed no significant difference in pain intensity between tDCS and sham stimulation (SMD = −0.22, 95% CI [−0.52, 0.08], Z = 1.44, *p* = 0.15).

Across the five included studies, seven trials targeted four distinct cortical regions: one trial targeted the DLPFC [16], one targeted the medial prefrontal cortex (mPFC) [28], one targeted the dorsal anterior cingulate cortex (dACC) [30], and two targeted M1 [15,29]. Subgroup meta-analysis was not feasible, as there were fewer than three trials for each stimulation target.

Narrative synthesis showed that a standalone of tDCS, regardless of the stimulation target, did not yield analgesic effects superior to sham stimulation (Table 1).

#### 3.3.2. Effect of Combined tDCS Interventions on Pain Intensity

**Figure 4:**
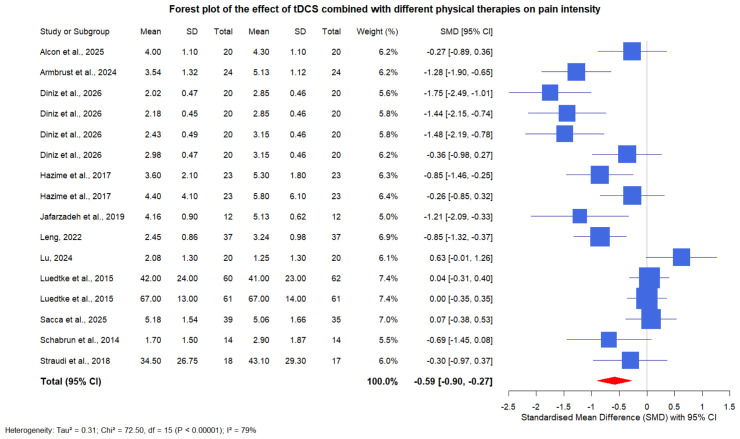
Forest plot of the effect of tDCS combined with different physical therapies on pain intensity [13,14,19,20,31,32,33,34,35,36,37]. Blue squares denote the SMD of individual studies, with horizontal lines representing 95% confidence intervals; the size of each square corresponds to study weight. The red diamond indicates the pooled overall SMD and its 95% CI.

A total of 11 randomized controlled trials [13,19,20,31,32,33,34,35,36,37] with 859 participants (tDCS: 431; control: 428) were included in the analysis (Figure 4). Using a random-effects model, the pooled results demonstrated that tDCS combined with various physical therapies significantly reduced pain intensity compared with control conditions (SMD = −0.59, 95% CI [−0.90, −0.27], Z = 3.65, *p* = 0.0003).

**Figure 5:**
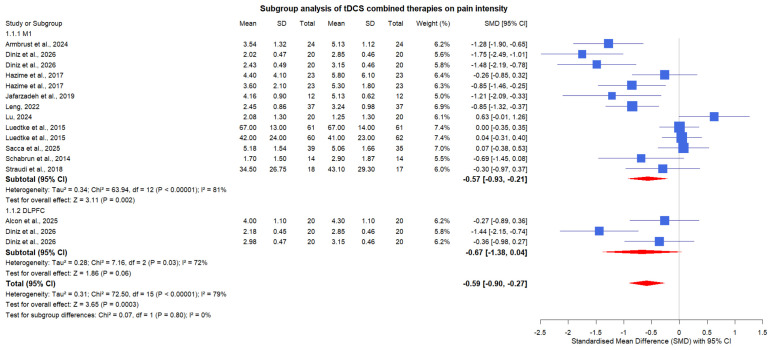
Subgroup analysis of tDCS combined therapies on pain intensity [13,14,19,20,31,32,33,34,35,36,37]. Blue squares denote the SMD of individual studies, with horizontal lines representing 95% confidence intervals; the size of each square corresponds to study weight. The red diamond indicates the pooled overall SMD and its 95% CI.

In the M1 subgroup, which included 11 studies [13,19,31,32,33,34,35,36,37], combined interventions such as exercise therapy, acupuncture, peripheral electrical stimulation, transcutaneous electrical nerve stimulation, osteopathic manipulative treatment, and cognitive behavioral management were analyzed (Figure 5) (Table 2). The results showed that M1-targeted tDCS combined with these therapies significantly reduced pain intensity (SMD = −0.57, 95% CI [−0.93, −0.21], Z = 3.11, *p* = 0.002), with high within-subgroup heterogeneity (I^2^ = 81%). The DLPFC subgroup comprised only two studies [20,37] and did not demonstrate a statistically significant analgesic effect (SMD = −0.67, 95% CI [−1.38, 0.04], Z = 1.86, *p* = 0.06), with high heterogeneity (I^2^ = 72%). A random-effects model showed no statistically significant difference between the M1 and DLPFC subgroups (Chi^2^ = 0.07, *p* = 0.80) (Figure 5).

#### 3.3.3. Effects of tDCS on Pain Threshold

Three studies assessed pressure pain threshold [13,20,28]. Alcon et al. [20] and McPhee et al. [28] found that neither tDCS nor tDCS combined with pain neuroscience education altered the pressure pain threshold. Schabrun et al. [13] reported that only tDCS combined with peripheral electrical stimulation (PES) increased the local pressure pain threshold, while tDCS, PES, or sham were ineffective. One additional study by McPhee et al. [28] evaluated conditioned pain modulation, temporal summation, and cuff algometry, reporting no significant between group differences in descending pain inhibition, central sensitization markers, or deep tissue pain sensitivity following HD-tDCS.

### 3.4. Effect of tDCS on Functional Disability

#### 3.4.1. Effect of Standalone tDCS on Functional Disability

**Figure 6:**
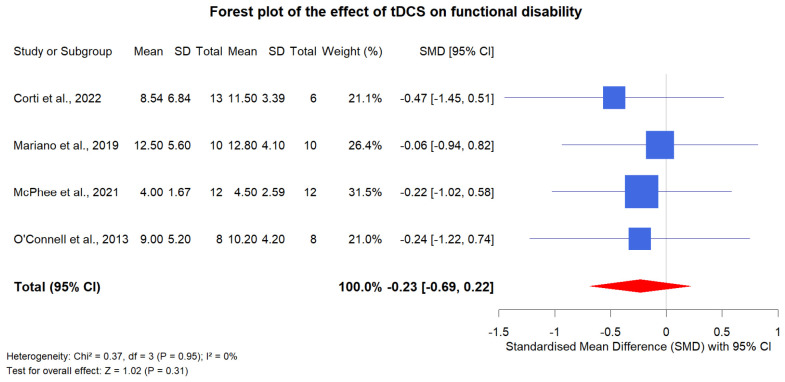
Forest plot of the effect of tDCS on functional disability [15,16,28,30]. Blue squares denote the SMD of individual studies, with horizontal lines representing 95% confidence intervals; the size of each square corresponds to study weight. The red diamond indicates the pooled overall SMD and its 95% CI.

Four randomized controlled trials [15,16,28,30] with a total of 79 participants (tDCS: 43; sham: 36) were included in the analysis (Figure 6). Using a fixed effects model, the pooled results showed that tDCS, regardless of stimulation target, did not significantly improve functional disability compared with sham stimulation (SMD = −0.23, 95% CI [−0.69, 0.22], Z = 1.02, *p* = 0.31) (Table 3).

#### 3.4.2. Effect of Combined tDCS Interventions on Functional Disability

**Figure 7:**
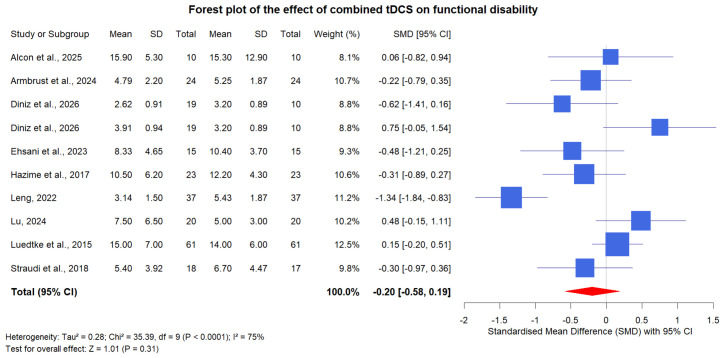
Forest plot of the effect of combined tDCS on functional disability [14,19,20,31,32,35,36,37,38]. Blue squares denote the SMD of individual studies, with horizontal lines representing 95% confidence intervals; the size of each square corresponds to study weight. The red diamond indicates the pooled overall SMD and its 95% CI.

A total of nine randomized controlled trials [14,19,20,31,32,35,36,37,38] with 473 participants (tDCS: 246; control: 227) were included in the analysis (Figure 7). Due to substantial heterogeneity across studies (I^2^ = 75%), a random effects model was applied. The pooled results showed no significant improvement in functional disability with combined tDCS interventions (SMD = −0.20, 95% CI [−0.58, 0.20], Z = 1.01, *p* = 0.31).

**Figure 8:**
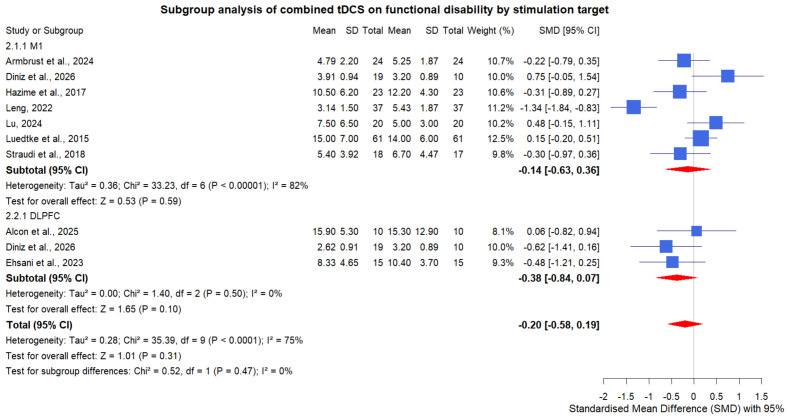
Forest plot of subgroup analysis of combined tDCS on functional disability [14,19,20,31,32,35,36,37,38]. Blue squares denote the SMD of individual studies, with horizontal lines representing 95% confidence intervals; the size of each square corresponds to study weight. The red diamond indicates the pooled overall SMD and its 95% CI.

Subgroup analyses stratified by stimulation target showed no statistically significant difference between the M1 and DLPFC subgroups (Chi^2^ = 0.52, *p* = 0.47) (Figure 8). In the M1 subgroup, which included seven trials [14,19,31,32,35,36,37] with 402 participants, combined tDCS did not significantly improve functional disability (SMD = −0.14, 95% CI [−0.63, 0.36], Z = 0.53, *p* = 0.59), with high within-subgroup heterogeneity (I^2^ = 82%). In the DLPFC subgroup, comprising three trials [20,37,38] with 88 participants, combined tDCS did not significantly improve functional disability (SMD = −0.38, 95% CI [−0.84, 0.07], Z = 1.65, *p* = 0.10) (Table 4).

### 3.5. Meta-Regression

To further explore potential sources of heterogeneity in the combined tDCS analysis, univariate meta-regressions were conducted with analgesic effect size (SMD) as the dependent variable and current intensity, total number of treatment sessions, and single session duration as independent variables. Neither current intensity nor total number of sessions showed significant moderator effects (coefficient = −0.54, *p* = 0.330, R^2^ = 0.068; coefficient = 0.03, *p* = 0.633, R^2^ = 0.017, respectively). Single session duration showed a statistically significant association (coefficient = −0.08, *p* = 0.025, R^2^ = 0.310), suggesting that longer stimulation duration may be associated with greater analgesic effects. This finding warrants particularly cautious interpretation, however. The vast majority of included studies employed sessions lasting 20 min (*n* = 15), while only two studies employed sessions lasting 30 min. Given this severe imbalance in data distribution, it is highly plausible that the observed association is confounded by other characteristics of the two studies employing longer sessions—such as the type or intensity of co-interventions, participant characteristics, or study design features—rather than reflecting a genuine effect of stimulation duration per se. In contrast, current intensity and total session number did not emerge as significant moderators, which is consistent with the low between-study variability in these parameters. Overall, these meta-regression findings suggest that protocol parameters alone cannot adequately explain the observed heterogeneity, indirectly supporting the interpretation that clinical diversity, particularly the type of concomitant peripheral intervention, constitutes the primary source of between-study variability. The detailed results of the meta-regression analyses are presented in Table S2.

### 3.6. Effect of tDCS on Psychological Outcomes

Due to the heterogeneity in the psychological constructs assessed across different studies and the variety of scales employed, coupled with the limited number of studies available for each specific outcome measure, a quantitative meta-analysis was precluded. Therefore, a descriptive synthesis is provided in this section to collate and summarize the evidence regarding the effects of tDCS on psychological outcomes in patients with chronic low back pain (Table 5).

#### 3.6.1. Pain Catastrophizing

Three studies assessed pain catastrophizing using the Pain Catastrophizing Scale [16,20,36]. In terms of combined interventions, Alcon et al. [20] reported that tDCS combined with pain neuroscience education enhanced improvements in pain catastrophizing; whereas Corti et al. [16] and Lu et al. [36] found no significant improvement.

#### 3.6.2. Kinesiophobia and Pain-Related Anxiety

Two studies assessed kinesiophobia using the Tampa Scale for Kinesiophobia [20,38], and two studies assessed pain-related anxiety using the Pain Anxiety Symptoms Scale [30,38]. Ehsani et al. [38] reported that tDCS combined with cognitive behavioral therapy significantly improved both outcomes; whereas Alcon et al. [20] found no additional benefit for kinesiophobia when tDCS was combined with Pain Neuroscience Education, and Mariano et al. [30] found no significant improvement in pain-related anxiety with standalone tDCS.

#### 3.6.3. Pain Acceptance

Only one study assessed pain acceptance [29], and it reported no significant improvement following standalone tDCS intervention.

#### 3.6.4. Other Psychological Measures

Several studies assessed other psychological outcomes using various scales. Luedtke et al. [14] reported that tDCS combined with cognitive behavioral management was ineffective for fear-avoidance beliefs, anxiety, and depression. For depressive symptoms, Straudi et al. [19] and Leng [35] found that tDCS combined with group exercise was effective, and Mariano et al. [30] found that standalone tDCS was effective. In contrast, Lu et al. [36] reported that tDCS combined with exercise was ineffective for depression and anxiety. Mariano et al. [30] also found that standalone tDCS was ineffective for generalized anxiety symptoms. For anxiety and emotional states, McPhee et al. [28] reported that HD-tDCS targeting the medial prefrontal cortex was ineffective for state anxiety, trait anxiety, positive affect, negative affect, and self-rated mood.

### 3.7. Effects of tDCS on Other Outcome Measures

Outcomes beyond pain and disability included sensorimotor, behavioral, cognitive, and neurophysiological/neurofunctional measures. Because few studies contributed data to most outcome domains and the assessment methods were heterogeneous, these findings were synthesized narratively.

#### 3.7.1. Effects of tDCS on Sensorimotor, Behavioral, and Somatosensory Outcomes

Findings for sensorimotor, behavioral, and somatosensory outcomes were mixed. In Jafarzadeh et al. [33], significant improvements were reported in both the Biodex stability indices and Berg Balance Scale scores. Schabrun et al. [13] found that only tDCS combined with PES significantly increased pain-free lumbar flexion. The same study also reported that both tDCS combined with PES and PES significantly reduced two-point discrimination thresholds, with no significant difference between these two interventions.

By contrast, electromyographic-based neuromuscular activation outcomes were largely null. In Diniz et al. [37], transversus abdominis activation did not differ across time points. Similarly, Jiang et al. [29] found no significant improvement in muscle activation patterns with tDCS.

#### 3.7.2. Effects of tDCS on Cognitive Outcomes

Evidence for cognitive outcomes was limited and inconsistent. Alcon et al. [20] reported that combined tDCS improved interference control and response inhibition, as measured by the Stroop Test and the Comprehensive Trail-Making Test-2 inhibition index, with large effect sizes; however, no between-group difference was observed for set-shifting. In contrast, O’Connell et al. [15] found no detectable between-group differences in cognitive task performance across multiple measures.

#### 3.7.3. Effects of tDCS on Neurophysiological and Neurofunctional Outcomes

Evidence for neurophysiological and neurofunctional outcomes showed modality-specific effects. Sacca et al. [34] reported condition-dependent changes in cerebral blood flow following both tDCS and sham tDCS, as well as both real and sham acupuncture.

### 3.8. Certainty of Evidence

The certainty of evidence as assessed by GRADE is summarized in Table S3. For pain intensity, the certainty of evidence was moderate for standalone tDCS and low for combined tDCS targeting M1. For the DLPFC subgroup, the certainty was very low. For functional disability, the certainty was moderate for standalone tDCS, and very low for combined tDCS targeting both M1 and DLPFC. Overall, the certainty of evidence ranged from moderate to very low, with the majority of comparisons rated as low or very low.

## 4. Discussion

This systematic review and meta-analysis primarily evaluated whether combining tDCS with peripheral interventions yields greater clinical benefits than standalone tDCS in patients with CNSLBP. Based on 17 randomized controlled trials, the results showed a clear distinction between these two application modalities: standalone tDCS did not produce significant analgesic effects, whereas tDCS combined with peripheral interventions yielded a significant synergistic reduction in pain intensity. Subgroup analyses comparing stimulation targets showed that M1-targeted tDCS significantly reduced pain within its subgroup, whereas DLPFC-targeted tDCS did not reach statistical significance; however, the difference between subgroups was not significant. The current data therefore do not suggest that one stimulation target is superior to the other. Beyond pain relief, combined interventions showed potential benefits for pain catastrophizing and postural control, yet these improvements did not translate into significant reductions in functional disability or changes in lumbar muscle activity.

The observed synergistic effect of combined tDCS interventions may be explained by state-dependent plasticity [39]. tDCS modulates cortical excitability through subthreshold membrane polarization, yet its ability to induce lasting neuroplastic changes—particularly those capable of reversing maladaptive pain networks—is thought to depend critically on concurrent afferent input. According to Hebbian plasticity theory [40], synchronous activation of cortical circuits strengthens synaptic connections. When tDCS is applied immediately before or during peripheral sensorimotor training, it may prime the motor cortex or prefrontal regions, thereby facilitating the consolidation of adaptive motor patterns and descending pain inhibitory pathways [41,42]. This framework provides a plausible explanation for why combined interventions produce synergistic analgesic effects whereas standalone tDCS does not. It should be noted, however, that this mechanistic account remains speculative, as the studies included in this meta-analysis did not directly assess neurophysiological markers of plasticity. The heterogeneous results reported in previous meta-analyses [43,44,45] may partly stem from pooling diverse protocols without distinguishing between standalone and combined applications, underscoring the importance of modality-stratified analyses. Regarding stimulation targets, although the M1 subgroup showed a statistically significant analgesic effect while the DLPFC subgroup did not, the between-subgroup difference was not significant. Critically, the DLPFC subgroup included only two studies, which substantially limits statistical power and inflates the risk of a Type II error. Notably, the point estimate for DLPFC stimulation was numerically larger than that for M1 stimulation, suggesting that the nonsignificant result for DLPFC likely reflects insufficient statistical power rather than a genuine lack of efficacy. Therefore, no inference regarding the differential efficacy of M1 versus DLPFC stimulation can be drawn from the present data. Whether distinct stimulation targets confer differential therapeutic effects remains an open question that requires adequately powered head-to-head trials directly comparing M1 and DLPFC stimulation within the same study.

This meta-analysis showed a dissociation between pain relief and functional improvement. Specifically, although combined tDCS interventions significantly reduced pain intensity, they did not improve disability outcomes. Disability in CNSLBP is multifactorial, driven not only by pain intensity but also by maladaptive motor control, trunk muscle atrophy, fear-avoidance beliefs, and long-standing behavioral adaptations [46,47]. Electromyography measures showed no significant changes in lumbar muscle activity [48,49], suggesting that short-term tDCS—even when combined with peripheral interventions—does not directly correct aberrant motor control. Based on the present findings, tDCS may offer greater clinical benefit when used as an adjunct to peripheral rehabilitation rather than as a standalone intervention, although this interpretation should be considered preliminary given the low to very low certainty of evidence for combined protocols. Future research should extend treatment duration, intensify functional training, and prolong follow-up to determine whether extended protocols can bridge the gap between pain relief and functional recovery.

The clinical significance of the observed effect sizes warrants consideration in light of established minimal clinically important difference (MCID) thresholds for chronic low back pain. The pooled SMD of −0.59 for pain intensity in combined tDCS interventions corresponds to a moderate effect size by conventional benchmarks. For reference, the MCID for pain intensity on a 0–10 numerical rating scale has been estimated at approximately 2 points [50], consistent with empirical evidence in low back pain populations [51]. The MCID for the Oswestry Disability Index has been established at approximately 10 points [52]. Although the present meta-analysis cannot directly convert the pooled SMD to original scale units due to variability in outcome measures across studies, a moderate SMD is generally considered to correspond to a clinically meaningful, albeit modest, improvement. In contrast, the functional disability SMD of −0.20 represents a small effect size and likely falls below established MCID thresholds. This discrepancy may partly explain the observed dissociation between statistically significant pain relief and nonsignificant functional improvement: while combined tDCS may produce analgesic effects that approach or reach clinical relevance, these analgesic improvements alone may be insufficient to translate into meaningful functional gains. It is possible that targeted physical rehabilitation directed at movement retraining and muscular reconditioning is necessary to achieve functional recovery, but this hypothesis requires direct empirical testing.

Beyond pain intensity, this review comprehensively assessed multidimensional outcomes including psychological status, postural control, and lumbar muscle activity, providing a more complete picture of tDCS efficacy. In addition, the inclusive search strategy incorporating both English and Chinese databases captured a broader evidence base, addressing a gap in prior reviews limited to English language publications.

Although the limited number of studies per co-intervention category precludes formal statistical comparison, descriptive examination of the included trials reveals several noteworthy patterns. Among the 11 studies that combined tDCS with peripheral interventions targeting M1, the cointerventions included exercise therapy (k = 3), peripheral electrical stimulation or TENS (k = 3), osteopathic manipulative treatment (k = 1), acupuncture (k = 1), cognitive behavioral management (k = 1), pain neuroscience education (k = 1), and postural training (k = 1). Studies combining tDCS with active movement-based therapies, such as exercise therapy and postural training, tended to report larger analgesic effects than those combining tDCS with passive modalities. However, these observations are preliminary and are confounded by differences in tDCS parameters, participant characteristics, and study quality across trials. Future research employing factorial designs that systematically manipulate both the type of cointervention and tDCS parameters is needed to identify the most effective combination strategies.

In the present meta-analysis, substantial statistical heterogeneity was observed in the overall analysis of combined tDCS interventions (I^2^ = 75–81%). To explore potential sources of this heterogeneity, subgroup analyses stratified by stimulation target were conducted. Within the M1 subgroup, heterogeneity remained high (I^2^ = 81%), whereas the DLPFC subgroup also showed considerable heterogeneity (I^2^ = 72%), albeit based on only two studies. These findings suggest that stimulation target alone does not fully account for the observed variability. Notably, as summarized in Table 2, the number and frequency of tDCS sessions varied considerably across studies, ranging from 4 to 12 sessions delivered either in consecutive daily sessions or at a frequency of three times per week. Such differences in cumulative stimulation dose may influence the magnitude of analgesic effects and could represent an important source of between-study variability. This is consistent with evidence from a recent meta-analysis of tDCS for orthopedic pain, in which the effectiveness of tDCS varied according to the number of treatment sessions [44], and with meta-regression findings in fibromyalgia showing that longer tDCS protocols (≥4 weeks) were associated with larger effect sizes [53]. Early work further showed that repeated daily sessions of anodal tDCS over M1 produced cumulative and sustained pain relief, supporting the concept that cumulative stimulation dose contributes to analgesic outcomes. In addition, differences in the type of cointerventions, variation in other tDCS parameters, and differences in participant characteristics likely further contributed to the heterogeneity. Due to the limited number of studies in each category, further subgroup analyses or meta-regression by session number, frequency, or cointervention type were not feasible. This limitation is acknowledged, and future studies with more standardized protocols are warranted to clarify the specific sources of heterogeneity.

The GRADE assessment showed that the overall certainty of evidence ranged from moderate to very low across the six evaluated comparisons. For pain intensity, moderate-certainty evidence from standalone tDCS trials was downgraded primarily due to imprecision arising from limited sample sizes. Low-certainty evidence for combined tDCS targeting M1 reflected serious concerns regarding inconsistency (I^2^ = 81%) and indirectness stemming from heterogeneous cointerventions. For the DLPFC subgroup, the evidence was of very low certainty owing to very serious imprecision, as only two studies with wide confidence intervals were available. For functional disability, the evidence was of moderate certainty for standalone tDCS but of very low certainty for both M1 and DLPFC combined protocols, driven by inconsistency, indirectness, and imprecision. These assessments indicate that the existing evidence base, while suggestive of potential analgesic benefits of combined tDCS protocols, is not yet sufficiently robust to support definitive clinical recommendations. These GRADE ratings are consistent with the observed patterns of heterogeneity and imprecision discussed above and reflect the current state of the evidence base for tDCS in CNSLBP.

Several limitations should be acknowledged. First, substantial clinical heterogeneity was present across the included studies, particularly regarding the types of cointerventions combined with tDCS, as well as variation in tDCS parameters including the number and frequency of sessions, current intensity, and electrode montage. Although subgroup analyses stratified by stimulation target were conducted to explore heterogeneity, residual within-subgroup heterogeneity remained high, indicating that target site alone does not fully account for the observed variability. Second, the number of studies in the DLPFC subgroup was small (two studies for pain intensity, three for functional disability), which substantially limits statistical power and inflates the risk of Type II error, resulting in very low certainty of evidence and precluding definitive conclusions regarding DLPFC specific efficacy. Third, publication bias could not be formally assessed via funnel plot asymmetry for most comparisons owing to the limited number of included studies per analysis. Fourth, the majority of included trials had short follow-up periods, and the durability of observed effects beyond the immediate postintervention period remains uncertain. Fifth, although a comprehensive set of outcomes was examined, the assessment of certain secondary domains—including quantitative sensory testing, cognitive function, and neurophysiological markers—was based on descriptive synthesis rather than quantitative meta-analysis due to the limited number of studies and heterogeneity of measurement tools, and these findings should be regarded as preliminary. Finally, only one included study employed HD-tDCS, precluding any comparison between conventional tDCS and HD-tDCS modalities [28]. Future research using HD-tDCS for CNSLBP is warranted, as its more focal current delivery may offer distinct advantages for targeting specific cortical regions.

## 5. Conclusions

Moderate-certainty evidence suggests that standalone tDCS does not provide clinically meaningful analgesic benefits for patients with CNSLBP. Low-certainty evidence indicates that combining tDCS with peripheral interventions—particularly anodal tDCS over M1 applied prior to or concurrently with structured exercise therapy—may produce synergistic analgesic effects; however, this finding should be interpreted with caution. Regarding stimulation targets, the available evidence does not demonstrate the superiority of M1 over DLPFC, or vice versa. These findings highlight the need for large-scale, high-quality randomized controlled trials with standardized protocols and extended follow-up to strengthen the evidence base.

## Figures and Tables

**Table 1:** Summary of tDCS Protocol for Pain Intensity.

Author	Number of Sessions	Stimulated Brain Region	Stimulation Montage	Current Intensity (mA)
Corti et al., 2022 [16]	8	DLPFC	Anodal stimulation	1.5
Mariano et al., 2019 [30]	10	dACC	Cathodal stimulation	2
McPhee et al., 2021 [28]	3	mPFC	Anodal stimulation	2
O’Connell et al., 2013 [15]	Average of 9 sessions per person ^1^	M1	Anodal stimulation	2
Jiang et al., 2019 [29]	1	M1	Anodal stimulation	2

^1^ In the study by O’Connell et al., 2013 [15], all participants first received sham stimulation (1) and were then switched to daily active stimulation at a randomly assigned time point (between days 1–15) until the end of the experimental period. The average number of active stimulation sessions per person was 9 (range: 3–14).

**Table 2:** Summary of tDCS protocol for pain intensity.

Author	Number of Sessions and Frequency	Stimulated Cortical Target	Modality	Intensity (mA)	Stimulation Duration (min)
Armbrust et al., 2024 [31]	10 consecutive workdays, 1 session per day, total of 10 sessions	M1	Anodal	2	20
Hazime et al., 2017 [32]	3 times/week, total of 4 weeks, total of 12 sessions	M1	Anodal	2	20
Jafarzadeh et al., 2019 [33]	3 times/week, total of 2 weeks, total of 6 sessions	M1	Anodal	2	20
Luedtke et al., 2015 [14]	5 consecutive days, 1 session per day, total of 5 sessions	M1	Anodal	2	20
Sacca et al., 2025 [34]	Total of 6 sessions	M1	Anodal	2	20
Schabrun et al., 2014 [13]	Total of 4 sessions, each intervention type once	M1	Anodal	1	30
Straudi et al., 2018 [19]	1 session per day for 5 consecutive days	M1	Anodal	2	20
Leng, 2022 [35]	1 session per day for 5 consecutive days, total of 5 sessions	M1	Anodal	2	20
Lu et al., 2024 [36]	3 times/week, total of 4 weeks, total of 12 sessions	M1	Anodal	2	20
Diniz et al., 2026 [37]	10 consecutive workdays, 1 session per day, total of 10 sessions	M1	Anodal	2	30
		DLPFC			
Alcon et al., 2025 [20]	Completed all sessions within 2 weeks, total of 5 sessions	DLPFC	Anodal	2	20

Note: DLPFC is the dorsolateral prefrontal cortex; M1 is the primary motor cortex.

**Table 3:** Summary of tDCS protocol for functional disability.

Author	Number of Sessions and Frequency	Stimulated Cortical Target	Modality	Intensity (mA)	Stimulation Duration (min)
Corti et al., 2022 [16]	2 times/week, total of 8 sessions	DLPFC	Anodal	1.5	20
Mariano et al., 2019 [30]	10 consecutive workdays, 1 session per day, total of 10 sessions	dACC	Cathodal	2	20
McPhee et al., 2021 [28]	3 consecutive days, 1 session per day, total of 3 sessions	mPFC	Anodal	2	20
O’Connell et al., 2013 [15]	Average 9 sessions	M1	Anodal	2	20

Note: dACC is the dorsal anterior cingulate cortex; mPFC is the medial prefrontal cortex.

**Table 4:** Summary of tDCS protocol for functional disability.

Author	Number of Sessions and Frequency	Stimulated Cortical Target	Modality	Intensity (mA)	Stimulation Duration (min)
Armbrust et al., 2024 [31]	10 consecutive workdays, 1 session per day, total of 10 sessions	M1	Anodal	2	20
Hazime et al., 2017 [32]	3 times/week, total of 4 weeks, total of 12 sessions	M1	Anodal	2	20
Luedtke et al., 2015 [14]	5 consecutive days, 1 session per day, total of 5 sessions	M1	Anodal	2	20
Straudi et al., 2018 [19]	1 session per day for 5 consecutive days	M1	Anodal	2	20
Leng, 2022 [35]	1 session per day for 5 consecutive days, total of 5 sessions	M1	Anodal	2	20
Lu et al., 2024 [36]	3 times/week, total of 4 weeks, total of 12 sessions	M1	Anodal	2	20
Diniz et al., 2026 [37]	10 consecutive workdays (Monday to Friday), 1 session per day, total of 10 sessions	M1	Anodal	2	30
		DLPFC			
Alcon et al., 2025 [20]	Completed all sessions within 2 weeks, total of 5 sessions	DLPFC	Anodal	2	20
Ehsani et al., 2023 [38]	2 times/week, total of 4 weeks, total of 8 sessions	DLPFC	Cathodal	2	20

**Table 5:** Narrative synthesis of the effects of tDCS on pain-related psychological outcomes.

Author	Method	Psychological Assessment Tool	Main Findings
Corti et al., 2022 [16]	tDCS	PCS	Failed to effectively improve pain scores
Alcon et al., 2025 [20]	tDCS + PNE	PCS, TSK	Combined benefits only for pain catastrophizing
Ehsani et al., 2023 [38]	tDCS + CBT	PASS, TSK	No combined benefit shown
Lu et al., 2024 [36]	tDCS + Exercise	PCS	No combined benefit shown
Mariano et al., 2019 [30]	tDCS	PASS, CPAQ-8	Failed to effectively improve pain-related anxiety or pain acceptance

Note: PCS is the Pain Catastrophizing Scale, TSK is the Tampa Scale for Kinesiophobia, PASS is the Pain Anxiety Symptoms Scale, CPAQ-8 is the Chronic Pain Acceptance Questionnaire-8.

## Data Availability

The data are available on request from the corresponding author.

## Supplementary Materials

The following supporting information can be downloaded at: https://www.mdpi.com/article/10.3390/healthcare14121764/s1, Table S1: Search strategy for databases. Table S2: Meta-Regression. Table S3: GRADE Summary of tDCS Effects on Pain and Disability.
