# Supplementary material for: Efficacy of Transcranial Direct Current Stimulation for Chronic Non-Specific Low Back Pain: A Systematic Review and Meta-Analysis
Source: Healthcare (Basel). 2026 Jun 18;14(12):1764. doi: 10.3390/healthcare14121764 (PMC13300147; doi:10.3390/healthcare14121764)
Supplement: Supplementary file 1 [file healthcare-14-01764-s001.zip › healthcare-4205851-supplementary.pdf]

Supplementary Material

Table S1 Search strategy for databases

**PubMed**	((((("Low Back Pain"[Mesh]) OR (chronic low back pain)) OR (chronic nonspecific low back pain)) OR (LBP)) OR (NSLBP)) AND ((((((("Transcranial Direct Current Stimulation"[Mesh]) OR (tACS)) OR (tRNS)) OR (tPCS)) OR (HD-tDCS)) OR (CES)) OR (Transcranial Electrical Stimulation))
**Web of Science**	TS=((("low back pain" OR "chronic low back pain" OR "chronic nonspecific low back pain" OR LBP OR nsabp) AND ("transcranial direct current stimulation" OR tDCS OR tACS OR tRNS OR tPCS OR "HD-tDCS" OR CES OR "transcranial electrical stimulation")))
**ScienceDirect**	TITLE-ABSTR-KEY(("low back pain" OR "chronic low back pain" OR LBP OR NSLBP OR "chronic nonspecific low back pain") AND ("transcranial direct current stimulation" OR tDCS OR tACS OR tRNS))
**Chinese Quarterly Virtual Information Platform (CQVIP)**	(((((((题名或关键词=慢性腰痛 OR 题名或关键词=chronic low back pain) OR 题名或关键词=chronic lumbago) OR 题名或关键词=chronic lumbar pain) OR 题名或关键词=慢性下背痛) OR 题名或关键词=慢性腰背痛) OR 题名或关键词=慢性下腰痛) AND ((((题名或关键词=tDCS OR 题名或关键词=tACS) OR 题名或关键词=tRNS) OR 题名或关键词=经颅直流电刺激) OR 题名或关键词=电刺激))
**China National Knowledge Infrastructure（CNKI）**	(主题:经颅电刺激 +经颅电刺激疗法)OR(篇关摘:经颅直流电刺激 +经颅直流电刺激治疗 + tACS + tRNS(精确))AND(主题:慢性腰痛 + 慢性腰痛患者 +'慢性腰痛(clbp)'+ 非特异性慢性腰痛)
**WANFANG DATA**	（（主题:(慢性腰痛) or 题名或关键词:(腰痛 or 非特异性腰痛)） and （主题:(经颅电刺激) or 题名或关键词:(经颅直流电刺激 or 经颅脉冲电流刺激 or 随机噪声刺激 or 经颅交流电刺激))

Table S2 Meta-Regression

Moderator variable	Coefficient	95% CI	P-value	R^2
**Current intensity (mA)**	−0.54	[−1.63, 0.55]	0.330	0.068
**Total number of sessions**	0.03	[−0.09, 0.14]	0.633	0.017
**Single-session duration (min)**	−0.08	[−0.16, −0.01]	0.025	0.310

Table S3 GRADE Summary of tDCS Effects on Pain and Disability

Outcome	Comparison	No. of studies (participants)	Effect estimate (SMD, 95% CI)	I^2	Limitations	Certainty
Pain intensity	Standalone tDCS vs sham	5 (91)	-0.22 [-0.52, 0.08]	0%	Imprecision (-1)	Moderate
	Combined tDCS (M1) vs control	11 (371)	-0.57 [-0.93, -0.21]	81%	Inconsistency (-1); Indirectness (-1)	Low
	Combined tDCS (DLPFC) vs control	2 (60)	-0.67 [-1.38, 0.04]	72%	Very serious imprecision (-2)	Very low
Functional disability	Standalone tDCS vs sham	4 (43)	-0.23 [-0.69, 0.22]	0%	Imprecision (-1)	Moderate
	Combined tDCS (M1) vs control	7 (202)	-0.12 [-0.63, 0.38]	82%	Inconsistency (-1); Indirectness (-1); Imprecision (-1)	Very low
